# Postoperative delirium, neuroinflammation, and influencing factors of postoperative delirium: A review

**DOI:** 10.1097/MD.0000000000032991

**Published:** 2023-02-22

**Authors:** M. Z. Xiao, C. X. Liu, L. G. Zhou, Y. Yang, Y. Wang

**Affiliations:** a Department of Anesthesiology, The Second Affiliated Hospital of University of South China, Hengyang, China; b Department of Anatomy, Hengyang Medical College of University of South China, Hengyang, China.

**Keywords:** cognitive impairment, microglial cell, neuroinflammation, postoperative delirium, synaptic plasticity

## Abstract

Postoperative delirium (POD) is an acute cognitive dysfunction that is mainly characterized by memory impairment and disturbances in consciousness. POD can prolong the hospital stay and increase the 1-month mortality rate of patients. The overall incidence of POD is approximately 23%, and its prevalence can go up to 50% in high-risk surgeries. Neuroinflammation is an important pathogenic mechanism of POD that mediates microglial activation and leads to synaptic remodeling. Neuroinflammation, as an indispensable pathogenesis of POD, can occur due to a variety of factors, including aseptic inflammation caused by surgery, effects of anesthetic drugs, disruption of the blood-brain barrier, and epigenetics. Understanding these factors and avoiding the occurrence of risk factors may help prevent POD in time. This review provides a brief overview of POD and neuroinflammation and summarizes various factors affecting POD development mediated by neuroinflammation, which may serve as future targets for the prevention and treatment of POD.

## 1. Introduction

Postoperative delirium (POD) is a serious post-surgical complication of the central nervous system (CNS). It mainly manifests as postoperative disturbances in consciousness, cognitive dysfunction, and impairment of the sleep-wake cycle.^[1,2]^ POD generally occurs 2 to 5 days after surgery, and prolongs the hospital stay of patients by 2 to 3 days, leading to an increase of the 1-month mortality rate by 7 to 10%.^[3]^ According to statistics, the overall incidence of POD is approximately 23%, and the prevalence is 50% in high-risk surgeries, such as hip fractures and cardiac surgery, reaching 20% in the elderly population (>60 years).^[4]^ In addition, it has been suggested that POD may be an early expression of Alzheimer disease (AD) and that there may be a common pathogenesis for both ailments.^[5]^ Multiple mechanisms contribute to the development of POD, including neuroinflammation, neurotransmitter imbalance, altered biological rhythms, altered brain metabolism, and impaired neuronal network connectivity. Among these, the role of neuroinflammation in POD may have been underestimated. Numerous studies have shown that neuroinflammation plays an important role in POD development. Aseptic inflammation in the periphery of surgery activates the innate immune system and initiates the inflammatory process, ultimately leading to POD.^[6–8]^ This review briefly introduces the role of neuroinflammation in POD and summarizes the effects of surgery, blood-brain barrier (BBB), inflammatory factors and pathways, and anesthetic drugs on POD.

## 2. Methods

We searched for relevant research articles in PubMed from 2000 to 2022 using the keywords POD combined with neuroinflammation. The search included clinical trials, primary research, reviews, and original articles. We selected relevant articles based on the content of the manuscript; further, some research articles on epigenetics were included. Grey literature and non-English articles were excluded from the analysis. Finally, a total of 99 articles were included.

## 3. Results

### 3.1. POD

The word “delirium” derives from the Latin “delirare,” which means “to get out of the ravine,” that is, to deviate from a straight line and become insane.^[9]^ POD is a severe neuropsychiatric syndrome characterized by acute postoperative episodes of attention and other cognitive deficits. A recent study found that the probability of POD developing into long-term postoperative cognitive dysfunction (POCD) after 3 months is approximately 10%, and perioperative POD and POCD are collectively referred to as perioperative neurocognitive disorders.^[10]^ In addition, POD may eventually develop into AD. A decrease in the ratio of β-amyloid and Tau, a biomarker of AD, is associated with POD.^[5]^ This indicates that POD, a serious postoperative complication, will develop into more severe POCD and AD without timely intervention.

POD can be divided into 3 subtypes: hypoactive, hyperactive, and mixed.^[11]^ The diagnosis of POD consists of 2 steps. First, a direct assessment of the patient’s level of attention and arousal is performed at the bedside. Second, an indirect assessment is sought from the patient’s family members, medical staff, and medical records to determine whether the patient has acute mental and behavioral abnormalities.^[1]^ More than 50 tools are available for diagnosing POD. The Confusion Assessment Method scale is the most commonly used method for evaluating POD, but its sensitivity is low.^[12,13]^ The Delirium Rating Scale-98 is a simplified version of DRS that provides an appropriate balance of specificity and sensitivity.^[14,15]^ The Delirium Observation Scale is a commonly used and accurate screening tool for the early identification of delirium. It is a short scale with 13 observations that is mainly completed by nurses and has a sensitivity of 90% and a specificity of 92%.^[16]^ In the case of hip fractures, the single-question delirium scale is often used, and if a patient’s score is positive, the 4 ‘A’s Test scale is implemented.^[17]^ The Confusion Assessment Method for the intensive care unit and Intensive Care Delirium Screening Checklist are the most effective and reliable tools for assessing POD in critically ill patients. In addition, the Stanford Proxy Test of Delirium and the Three-Minute Delirium Diagnostic Scale are commonly used to diagnose POD.^[18,19]^

**Figure 1.**
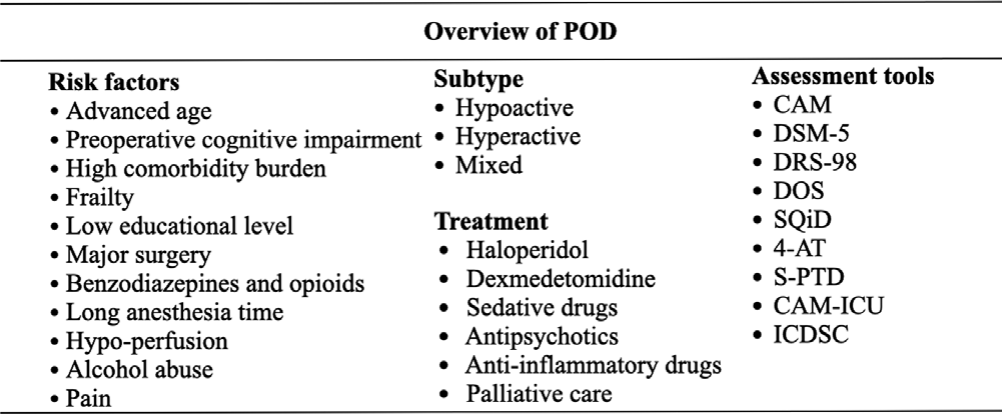
Prevention, diagnosis, and treatment of POD. POD = postoperative delirium.

Currently, there are no specific methods for the treatment of POD. Sedatives and antipsychotic drugs such as dexmedetomidine and haloperidol are mainly used in clinical palliative treatment. In addition, anti-inflammatory drugs have therapeutic value. Studies have found that a certain dose of dexamethasone administered during surgery can reduce the incidence of POD.^[20]^ Some nonsteroidal anti-inflammatory drugs, such as acetaminophen and parecoxib, have shown a protective effect against POD during the application of multimodal analgesia (Fig. 1).^[21,22]^

### 3.2. Neuroinflammation

Neuroinflammation refers to the peripheral inflammatory response triggered by surgery, trauma, or infection. It causes a large number of inflammatory mediators such as interleukin (IL)-6, IL-1β, and tumor necrosis factor-α (TNF-α) to pass through the BBB and induces damage to central neurons and synapses.^[23]^ Disruption of the BBB is thought to cause neuroinflammation.^[24]^ According to a murine tibial fracture surgery model, activation of T cells increases the levels of IL-17A, and disruption of the BBB is thought to be associated with POD-like behavior.^[25]^ Sometimes, the body has a systemic inflammatory response such as sepsis. Sepsis endows microglia with pro-inflammatory functions and microglia produce a series of pro-inflammatory and neurotoxic factors, thereby expanding the central inflammatory response and neuronal damage.^[26]^ In response to endotoxemia, astrocytes secrete chemotactic ligand factor 11. This leads to microglial migration and production of reactive oxygen species that impair learning and memory in the adult brain, resulting in hippocampal neuronal damage, behavioral changes, and memory impairment.^[27]^ In addition to triggering a more severe inflammatory response, endotoxemia also promotes damage to the BBB. Stubbs et al showed that vasogenic edema and white matter hyperintensities were present on magnetic resonance imaging in patients with sepsis-associated encephalopathy, indicating BBB disruption.^[28]^ Simultaneously, a systemic inflammatory response activates the toll-like receptor (TLR) 4/nuclear factor-k-gene binding pathway, altering the structure and function of tight junctions (a structure that makes up the BBB).^[29]^

Rat and mouse surgical models are commonly used to assess postoperative inflammatory responses and cognitive function, and orthopedic and open surgical models are the most frequently used.^[5,30,31]^ In addition, there have been studies using only neuroinflammatory models to evaluate cognitive function. Intraperitoneal or lateral ventricle injection of lipopolysaccharide (LPS) is a common technique used to simulate neuroinflammation in animal models. Several studies have shown that intraperitoneal or lateral injection of LPS can induce the infiltration of inflammatory factors into the brain, resulting in delirium-like behavioral changes in mice.^[32–35]^ In cell experiments, exogenous administration of LPS caused microglia to secrete a large number of inflammatory factors, such as IL-1β.^[36]^ Thus, IL-1β may play a key role in cognitive dysfunction. In the APP/PS1 mouse model, administration of IL-1β disrupted gamma network activity in the mouse hippocampus, impairing cognitive functions such as learning, memory, and executive abilities.^[37]^ Recruitment of IL-1β, monocytes, and neutrophils plays an important role in the occurrence and development of cognitive dysfunction. However, some studies have found that LPS-induced systemic inflammatory response is dependent on the IL-1 receptor, and the resulting neurotic electrophysiological hyperexcitability and neuronal death are mechanistically different from LPS-induced acute cognitive impairment.^[38]^ Scopolamine injection is another commonly used method to model POD and is based on the theory that systemic inflammatory responses are controlled by vagal-regulated cholinergic anti-inflammatory pathways.^[39]^ In a laparotomy mouse model, intraperitoneal injection of scopolamine resulted in the development of POD in mice.^[31]^

### 3.3. Neuroinflammation mediates factors influencing POD

**Figure 2.**
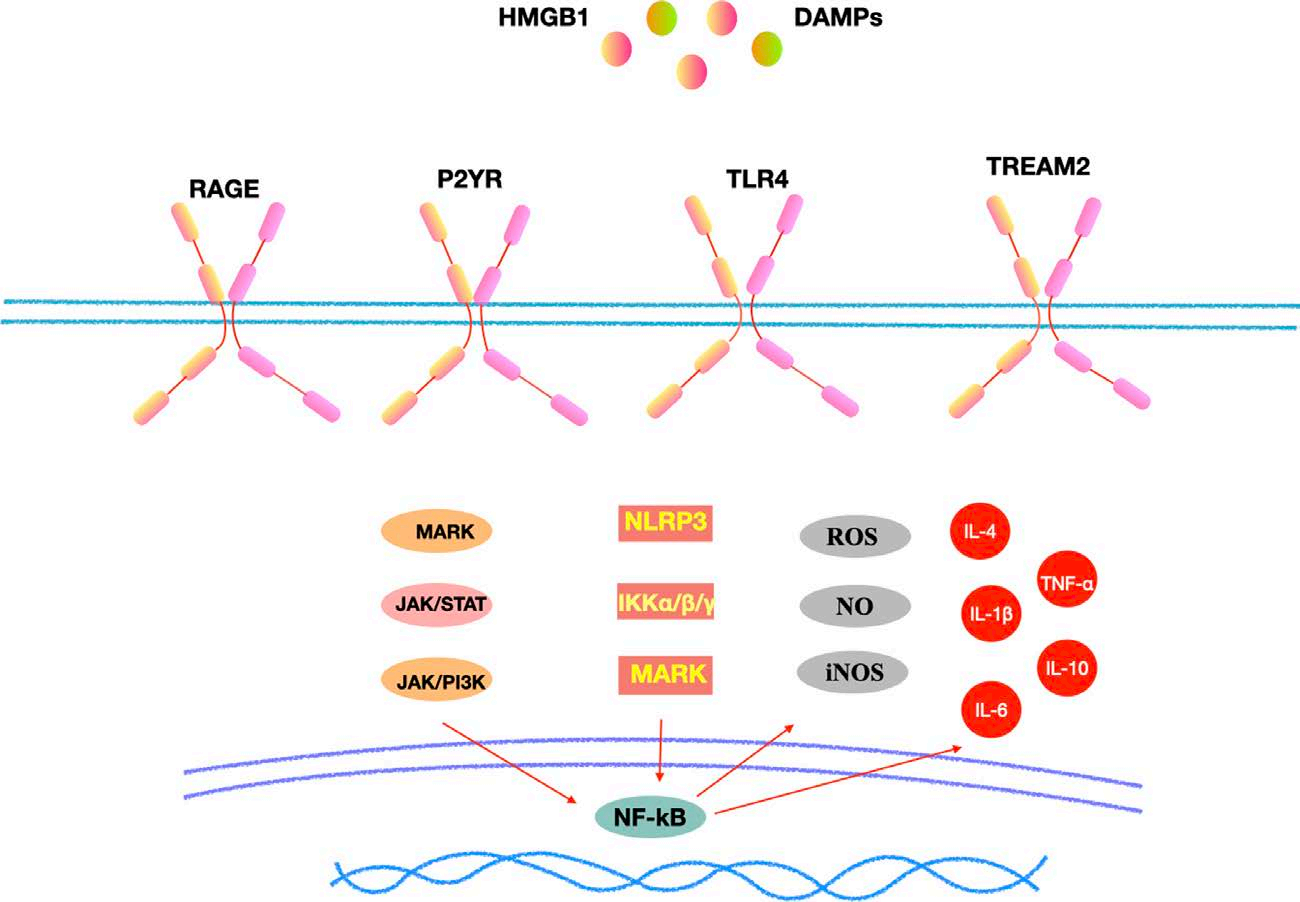
Neuroinflammatory pathway: Surgical trauma can induce central inflammation by upregulating the expression of HMGB1 and other DAMPs, activating MAPK/JAK/STAT, NLRP3/IKK, and other inflammatory pathways through RAGE, P2Y, TLR4, and TREM2 receptors, and releasing ROS, NO, IL-6, IL-1β, and other inflammatory mediators. DAMPs = damage-associated molecular patterns, HMGB1 = high mobility group box 1, IKK = IkappaB kinase, IL = interleukin, JAK = janus kinase, NLRP3 = NLR family pyrin domain containing 3, NO = nitric oxide, RAGE = receptor for advanced glycation end products, ROS = reactive oxygen species, STAT = signal transducer and activator of transcription, TLR4 = toll-like receptor 4, TREM2 = triggering receptor expressed on myeloid cells-2.

Regardless of which inflammatory pathway causes POD, neurons, and synapses are ultimately affected, resulting in changes in synaptic function. Currently, it is believed that neuroinflammation-mediated POD is mainly related to microglial activation. Microglia in the healthy CNS have highly branched processes at rest, but when activated, they become ameboid and are associated with phagocytic debris, antigens, and synaptic pruning.^[40]^ Damage to the BBB can cause microglial activation, and an anti-inflammatory and repair phase is rapidly initiated, which entails the polarization of microglia into the M1 and M2 phenotypes.^[41]^ M1 microglia play an immediate role in injury or infection and can produce a large number of pro-inflammatory factors, such as TNF-α, IL-1β, nitric oxide, and reactive oxygen species.^[42]^ M2 microglia are related to anti-inflammatory effects and tissue repair and are mainly used for anti-inflammatory factors, IL-4, IL-13, IL-10, and TGF-β to mitigate the inflammatory response.^[43]^ The polarization of microglial M1 and M2 phenotypes is only a theoretical outline, and they can induce acute inflammation and neuronal death in the process of injury repair, thereby impairing cognitive function. These factors may be involved in neuroinflammation-mediated POD (Fig. 2).

#### 3.3.1. Surgery.

The concept of brain immune privileges has recently been revised. Surgery causes sterile trauma, and the resulting cellular damage triggers endogenous factors called damage-associated molecular patterns.^[44]^ A prospective study of elderly patients undergoing tumor surgery found that surgery in itself can cause an increase in IL-10, IL-6, and IL-1β in the peripheral blood.^[14]^ Following surgical trauma, the innate immune system is activated in an NF-κB-dependent manner, leading to the release of multiple pro-inflammatory mediators and promoting the migration of monocyte-derived macrophages into the brain parenchyma.^[45]^ High mobility group box 1 is a typical damage-associated molecular pattern that is both a nuclear factor and a secreted protein and is involved in the regulation of various inflammatory processes.^[46]^ Soluble HBGB1 is also involved in the activation of multiple pattern recognition receptors, including TLR2, TLR4, and receptor for advanced glycation end products.^[44]^ In traumatic brain injury models, elevation of high mobility group box 1 and sustained activation of the NLR family pyrin domain containing 3 (NLRP3) inflammasome are thought to be key causes of traumatic brain injury-induced cognitive impairment.^[47]^ Intraoperative blood loss (>500 mL) and operative time (>3 hours) are also considered risk factors for POD, and intraoperative monitoring of cerebral oxygen saturation may be an important strategy to prevent POD.^[14]^

#### 3.3.2. Anesthetic.

Benzodiazepines, which act on gamma-aminobutyric acid (GABA) receptors, are closely related to cognitive function; however, their role in POD is controversial. Midazolam is often used for sedation of multiactivity delirium in intensive care unit patients with POD. A prospective study of noncardiac surgery showed that preoperative midazolam administration was not associated with the incidence of POD.^[48]^ Although the effect of midazolam on POD does not appear to be mediated by inflammatory signaling pathways, it may be related to cholinesterase genes.^[49]^ Recently, the use of a new benzodiazepine, remimazolam, was found to reduce the incidence of POD in cardiac surgery.^[50]^ Remimazolam, an ultrashort-acting benzodiazepine, ameliorated the LPS-induced peripheral blood septic response in mice, reduced the number of LPS-induced deaths, and decreased the production of inflammatory factors in cultured macrophages in vitro.^[51]^ In contrast, animal studies have shown that remimazolam can cause behavioral abnormalities and neuronal degeneration in mice.^[52]^

Dexmedetomidine, an adrenergic receptor agonist, has also been found to prevent POD. Dexmedetomidine has anti-inflammatory, antiarrhythmic, and myocardial perfusion-improving effects, and its anti-POD effects are widely recognized.^[53]^ The use of dexmedetomidine in geriatric hip fracture surgery reduces the incidence of POD.^[54]^ In contrast, esketamine affects the incidence of POD primarily by acting on n-methyl-d-aspartate receptors. Inhaled anesthetics and opioids are also considered risk factors for POD. In an animal model of open surgery, inhalation of isoflurane anesthesia damaged the BBB in mice, increasing the permeability of the BBB, and incidence of POD.^[55]^ In addition, pain influences inflammation and POD.^[53]^ Effective analgesia in the perioperative period has been found to help reduce neuroinflammation and delirium-like behavior.^[56]^

#### 3.3.3. BBB.

**Figure 3.**
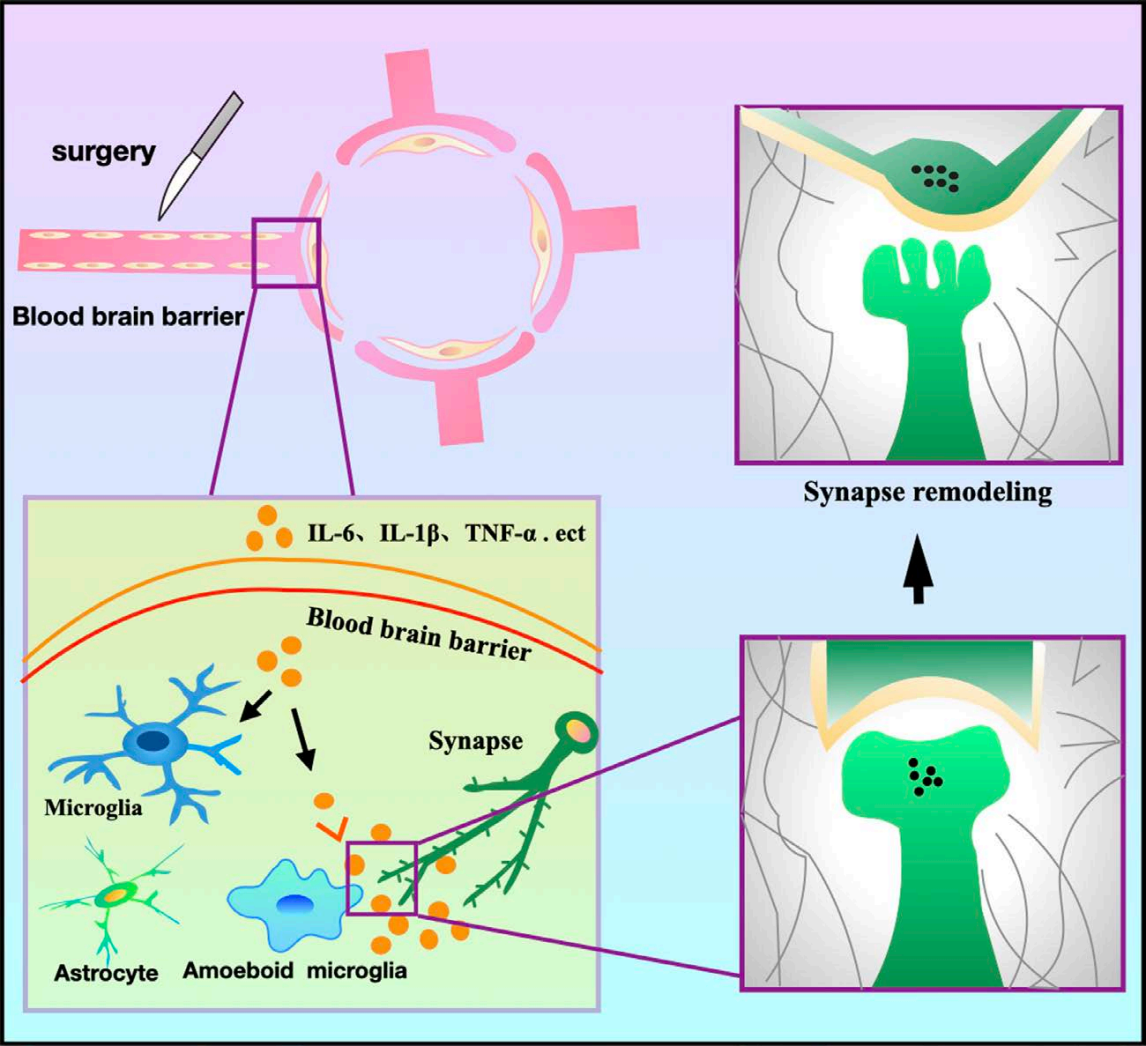
Neuroinflammation leads to synaptic remodeling: Surgery leads to aseptic inflammation in the periphery and the release of a large number of inflammatory cytokines resulting in increased BBB permeability. Inflammatory mediators enter the central nervous system from the periphery and activate microglia. The interaction between microglia and synapses can lead to synaptic remodeling and dysfunction. BBB = blood-brain barrier.

The BBB is formed by microvascular endothelial cells lining the cerebral capillaries. The induction and maintenance of barrier function depend primarily on interactions between the microvascular endothelium, astrocytic foot processes (which account for approximately 99% of the surface area of the brain capillary outer wall), and pericytes. Under pathological inflammatory conditions, the connections between endothelial cells are disrupted, leading to increased permeability of the BBB.^[57]^ Disruption of this barrier is the first step in neuroinflammation. A case-control study comparing cerebrospinal fluid-to-plasma albumin ratios and plasma S100β levels in POD patients with those in healthy controls found that POD was associated with BBB disruption and neuroinflammation.^[58]^ Following surgical trauma-induced activation of the innate immune system, inflammatory cytokines or macrophages from peripheral blood mononuclear cells diffuse passively into the brain through a compromised BBB.^[24,59]^ Inflammatory factors then enter the brain via active carrier-mediated transport through damaged BBB.^[24]^ Finally, peripheral inflammatory signals act on the afferent branches of the vagus nerve, activating microglia in the brain and inflammatory response, which leads to synaptic dysfunction and neuronal apoptosis, ultimately impairing cognitive function (Fig. 3).^[60]^

#### 3.3.4. Glial cells.

Microglial activation is a major component of CNS neuroinflammation and the first line of defense during injury or disease.^[41]^ Microglia are activated via various pathways. Activated microglia rapidly transform into a pro-inflammatory phenotype with an enlarged morphology and enhance the production of pro-inflammatory molecules.^[8]^ The pro-inflammatory cytokines and debris released by activated microglia can convert astrocytes into a neurotoxic A1 reactive subtype, causing them to lose normal synaptic maintenance and phagocytosis and induce rapid neuronal and oligodendrocyte death.^[41]^

In localized brain injury, microglia can clean up damaged brain tissue fragments and play a neuroprotective role by closing the gaps created by dead or damaged astrocytes and maintaining the integrity of the glial boundary barrier.^[61]^ Arg1 is a canonical marker of M2 macrophage/microglial activation and is involved in arginine metabolism. Arg1 is induced by IL-4 or IL-13 and acts as an anti-inflammatory agent by competitively inhibiting the substrate arginine and inhibiting nitric oxide production.^[62]^ TGF-β can play a reparative role by increasing the expression of Arg1 and Ym1 and enhancing the production of IL-4-induced M2 microglia.^[63]^ Microglia prune synapses via phagocytosis and regulate neuronal network activity.^[64]^ Inhibitory translocation of GABAergic pre-synapses by microglia increases synchronous firing in adult cortical neurons after exposure to LPS.^[64]^ The immune function of astrocytes is similar to that of microglia.^[65]^ Positron emission tomography imaging of neuroinflammation can be obtained using translocator protein imaging but is not as useful for differentiating microglial phenotypes or distinguishing between microglia and astrocytes.^[37,66]^ Co-culture of inflammatory supernatants containing high levels of IL-17A in astrocytes or activated astrocyte supernatants had significant neuroprotective effects.^[67]^ Poststroke astrocytes also promote tissue repair by producing IL-17A.^[68]^

#### 3.3.5. Inflammatory mediators and receptors.

Inflammatory factors such as IL-6, IL-1β, IL-10, and TNF-α have been reported to be elevated in neuroinflammation-mediated POD; however, their specific mechanisms have not been studied in-depth (Table 1).^[75]^ One study identified the mechanism by which inflammatory cytokines mediate cognitive impairment by increasing microglial phagocytosis of the extracellular matrix (ECM) through frequent contact with dendritic spines, thereby affecting memory in mice, in which IL-33 plays an important role.^[76]^ IL-17A is also implicated in the relationship between neuroinflammation and cognitive function.^[77]^ IL-17A neutralization directly abrogates neuroinflammation and memory impairment.^[77]^ In addition, IL-17A was found to be involved in the maintenance of short-term memory, and IL-17A deficiency decreased the plasticity of glutamatergic synapses, resulting in impaired long-term potentiation of the hippocampus.^[77]^ The increase in IL-17A concentration can promote the production of brain-derived neurotrophic factor (BDNF) in glial cells, and exogenous administration of IL-17A can rescue the synaptic and behavioral phenotypes of IL-17A-deficient animals.^[77]^ However, the co-culture of activated microglia with highly enriched developing cortical interneurons produced neuronal and synaptic metabolic dysfunction that could not be resolved by the exogenous addition of IL-17A, suggesting that IL-17A does not affect the metabolism of developing cortical interneurons.^[78]^

**Table 1:** Biological and inflammation markers of POD induced by operation.

Operation type	Biological markers	Sample	Trend	POD related	Reference
Oncologic surgery	IL-10, NGAL	Blood	Up	Yes	Brattinga et al 2022^[14]^
	IL-6, IL-1β, CRP	Blood	Up	No	
Hip fracture surgery	sTREM2	Cerebrospinal fluid	Up	Yes	K. Henjum et al 2018^[69]^
Hip fracture surgery	S100β	Blood	Up	No	Beishuizen et al 2017^[70]^
Abdominal surgery	TNF-α, TNF-R1, IL-6, IL-10, IL-1ra, CRP, SAA, NFL, Tau	Blood	Up	Yes/no	Anton Forsberg et al 2017^[71]^
Off-pump coronary artery bypass surgery	S100β	Blood	Up	Yes	Al Tmimi et al 2016^[72]^
Noncardiac surgery	IL-6	Blood	Up	Yes	Pei Liu et al 2013^[73]^
Coronary artery bypass surgery	NO3-/NO2-	Blood	Up	No	Harmon et al 2005^[74]^

CRP = C-reactive protein, IL = interleukin, NFL = neurofilament light chain, NGAL = neutrophil gelatinase-associated lipocalin, POD = postoperative delirium, SAA = serum amyloid A, TNF- α = tumor necrosis factor-α, TREM2 = triggering receptor expressed on myeloid cells-2.

The NLRP3 inflammasome is thought to be closely associated with altered cognitive function. NLRP3, an intracellular sensor that can detect a wide range of microbial substrates, has been shown to elevate the levels of pro-inflammatory cytokines IL-1β and IL-18 by activating caspase-1.^[79]^ In AD models, administration of the NLRP3 inhibitor Mcc950 attenuated Tau-induced IL-1β responses and reduced neuroinflammation as well as amyloid deposition associated with AD pathology.^[36]^ GABAAP is also thought to affect the NLRP3 inflammasome-dependent inflammatory response by mediating mitochondrial mass in macrophages.^[80]^

BDNF is a 13.5 kDa member of the neurotrophic factor protein family that affects neuroplasticity and neurotransmission and plays a key role in learning, memory, and cognition^.[81]^ BDNF is abundant in the CNS. It crosses the BBB, and BDNF levels in the blood correlate with BDNF levels in the cerebrospinal fluid and brain.^[81]^ Pro-inflammatory cytokines can inhibit BDNF signaling by activating p38 mitogen-activated protein kinase and nuclear factor-κB (NF-κB), resulting in reduced neurogenesis and neuroplasticity.^[82]^ However, BDNF levels are associated with neural network plasticity related to learning memory capacity and cannot be used as a blood marker of neuroinflammation.^[82]^

Peripheral benzodiazepine receptors 28 were found to be associated with long-term persistence of cognitive impairment after abdominal surgery.^[71]^ Peripheral benzodiazepine receptors 28 is a second-generation selective radiolabeled receptor for the 18 kDa translocator protein, also known as peripheral benzodiazepine receptor. It is a ubiquitously expressed transmembrane protein located outside the mitochondria of the microglial membrane and is also expressed in monocyte macrophages.^[83]^ Moreover, triggering receptor expressed on myeloid cells-2 has been implicated in aging and neurodegeneration. Triggering receptor expressed on myeloid cells-2 is an important innate immune receptor that signals through the adaptor protein TYRO protein tyrosine kinase binding protein/DAP12 and is expressed in microglia.^[84]^

#### 3.3.6. Purinergic pathway.

In recent years, the role of microglial purinergic receptors in neuroinflammation has been described, among which P_2_Y_12_R is selectively expressed in central microglia and regulates microglial morphology.^[83,85]^ Blockade of P_2_Y_12_R with clopidogrel prevents extensive microglia-neuron contact and presynaptic displacement.^[64]^ After the rupture of BBB, microglial chemotaxis via P_2_Y_12_R induces rapid healing of the BBB by forming dense aggregates at the injury site.^[86]^ A novel model of glial-neuron interaction, called microglial process convergence, proposes that excessive glutamate release activates neuronal n-methyl-d-aspartate receptors, triggering the release of the chemokine C-X3-C motif chemokine ligand 1 in neurons, which in turn activates microglial CX3CR1.^[69]^ CX3CR1 activation then induces the release of microglial IL-1β, which stimulates neuronal dendrites, subsequently triggering the release of ATP and acting on P_2_Y_12_R to induce local convergence of microglial processes.^[69]^ In addition, a study found that P_2_Y_1_R also plays a role in the migration of microglia.^[55]^

#### 3.3.7. Epigenetics.

Epigenetics refers to changes in gene expression caused by histone modification and DNA methylation in gene promoter regions. Increasing experimental evidence show that epigenetic signals play an important role in synaptic plasticity, learning, and memory effects. Studies have shown that inflammation in various tissues leads to changes in chromatin modification. For example, inflammation induces the aberrant trimethylation of histone 3 in mouse colonic epithelial cells.^[87]^ Histone methylation can inhibit the transcription of cytokines in mouse macrophages and protect against LPS-induced death.^[88]^ Tang et al found that trimethylation of histone 3 lysine 27 leads to an increased inflammatory phenotype in macrophages and microglia, whereas the histone 3 lysine 27 histone demethylase Jumonji domain-containing protein 3 is critical for promoting the anti-inflammatory M2 phenotype in microglia.^[54]^

Anesthetics may affect POD through epigenetics. Inhibition of the expression of DNA and histone-modifying enzymes by anesthetics can affect the methylation, histone acetylation, and histone methylation of epigenetic markers inflammatory factors such as IL-6, IL-1β, and TNF-α.^[11]^ Katharina et al reviewed epigenetic effects and found that the impact of anesthetics on DNA methylation appeared to be mixed.^[89]^ Zhang et al found that the blood folic acid levels decreased in children, which led to the downregulation of thymidylate synthase genes following the administration of sevoflurane. The main target of folate metabolism disorder is the Ermin-like protein, whose gene undergoes epigenetic variation after sevoflurane administration. Increased methylation of the Ermin-like protein promoter leads to decreased expression of Ermin-like proteins, resulting in brain demyelination and cognitive dysfunction.^[90]^ This suggests that Ermin-like proteins may be important targets of anesthesia through epigenetic mechanisms.

Inflammation and anesthetics can also influence histone acetylation. Histone acetylation and deacetylation are epigenetic processes mediated by histone acetyltransferases and deacetylases (HDACs). HDAC inhibitory activity can be found in drugs with known anti-inflammatory and neuroprotective functions, such as valproic acid.^[91]^ Lin et al showed that propofol application during early gestation could affect the learning and memory of offspring by inhibiting histone acetylation.^[92]^ A recent study showed that isoflurane anesthesia increased HDAC3 protein expression in the dorsal hippocampus of aged mice and decreased spinal dendrite density and levels of synaptic plasticity-related proteins.^[93]^

Several lines of evidence have also suggested a role for epigenetic mechanisms in BBB penetration and neuroinflammation. Katarzyna et al found that stress-induced BBB permeability is associated with endothelial inflammation and upregulation of the epigenetic repressor histone deacetylase 1 which reduces claudin-5 expression and may lead to the loosening of tight junctions and leakage of BBB.^[94]^ Claudin-5 is an integral membrane protein and an essential component of the tight junction protein complex that constitutes the BBB.^[95]^ Anke et al also showed that claudin-5 methylation was associated with cognitive impairment.^[96]^ IL-1β and TNF-α are strongly involved in BBB disruption through epigenetics.^[93]^ The BBB model in vitro releases IL-1β, which induces the degradation of occludin and zonula occludens-1 proteins by activating the ATP/P2X7R signaling pathway.^[97]^ TNF-α degrades occludin and promotes BBB damage through multiple signaling pathways. TNF-α induces phosphorylation in human brain endothelial cell lines, increases brain epithelial cell permeability, and disrupts the BBB through transient stimulation of the p38 mitogen-activated protein kinases and extracellular signal-regulated kinase 1/2 pathways.^[98]^

## 4. Discussion

As an acute cognitive impairment, POD not only severely affects the postoperative recovery of patients but can also have a great impact on families and society. Current research on neuroinflammatory mechanisms in POD is limited to a broad discussion of inflammatory factors and lacks specific indicators to diagnose or predict POD. Disruption of the BBB, activation of glial cells, and alterations in neuronal and synaptic functions are essential for neuroinflammation to mediate POD. However, the mechanisms of action and key factors that play a role, such as specific receptors, remain to be studied.

## 5. Conclusion

POD is a serious postoperative complication and neuroinflammation plays an important role in its pathogenesis. Inflammatory factors are measured in both the blood and cerebrospinal fluid, and future studies are expected to focus on the inflammatory signaling pathways for predicting POD and determining prognosis. Disruption of the BBB is a key step in neuroinflammation, and the effect of inflammation on BBB permeability and its mechanism needs to be studied further. Activation of glial cells is a symbol of neuroinflammation, but the molecular pathways that are involved remain unclear. Epigenetics plays a role in influencing POD and inflammation and maybe a new therapeutic target for POD. Other mechanisms may contribute to neuroinflammation-mediated POD, and further research is required to explore their relationships with POD for clinical prevention, diagnosis, and treatment.

## Acknowledgments

We thank members of the AJE and Wolters Kluwer Corporation for their comments and suggestions, and we would like to thank them for their English language editing.

## Author contributions

**Writing – original draft:** M. Z. Xiao, C. X. Liu, L. G. Zhou.

**Writing – review & editing:** Y. Yang, Y. Wang.

## References

[1] Wilson JE Mart MF Cunningham C. Delirium. Nat Rev Dis Primers. 2020;6:90. 33184265 10.1038/s41572-020-00223-4 PMC9012267

[2] Maldonado JR. Delirium pathophysiology: an updated hypothesis of the etiology of acute brain failure. Int J Geriatr Psychiatry. 2018;33:1428–57. 29278283 10.1002/gps.4823

[3] Jin Z Hu J Ma D. Postoperative delirium: perioperative assessment, risk reduction, and management. Br J Anaesth. 2020;125:492–504. 32798069 10.1016/j.bja.2020.06.063

[4] Bellelli G Brathwaite JS Mazzola P. Delirium: a marker of vulnerability in older people. Front Aging Neurosci. 2021;13:626127. 33994990 10.3389/fnagi.2021.626127 PMC8119654

[5] Subramaniyan S Terrando N. Neuroinflammation and perioperative neurocognitive disorders. Anesth Analg. 2019;128:781–8. 30883423 10.1213/ANE.0000000000004053 PMC6437083

[6] Girard S Brough D Lopez-Castejon G. Microglia and macrophages differentially modulate cell death after brain injury caused by oxygen-glucose deprivation in organotypic brain slices. Glia. 2013;61:813–24. 23404620 10.1002/glia.22478 PMC3644876

[7] Maldonado JR. Neuropathogenesis of delirium: review of current etiologic theories and common pathways. Am J Geriatr Psychiatry. 2013;21:1190–222. 24206937 10.1016/j.jagp.2013.09.005

[8] Saxena S Maze M. Impact on the brain of the inflammatory response to surgery. Presse Med. 2018;47(4 Pt 2):e73–81. 29656802 10.1016/j.lpm.2018.03.011 PMC6445379

[9] Adamis D Treloar A Martin FC. A brief review of the history of delirium as a mental disorder. Hist Psychiatry. 2007;18(72 Pt 4):459–69. 18590023 10.1177/0957154X07076467

[10] Brown CH. Delirium in the cardiac surgical ICU. Curr Opin Anaesthesiol. 2014;27:117–22. 24514034 10.1097/ACO.0000000000000061 PMC4156112

[11] Jackson TA Wilson D Richardson S. Predicting outcome in older hospital patients with delirium: a systematic literature review. Int J Geriatr Psychiatry. 2016;31:392–9. 26302258 10.1002/gps.4344

[12] Hshieh TT Inouye SK Oh ES. Delirium in the elderly. Clin Geriatr Med. 2020;36:183–99. 32222295 10.1016/j.cger.2019.11.001

[13] Heinrich TW Kato H Emanuel C. Improving the validity of nurse-based delirium screening: a head-to-head comparison of nursing delirium-screening scale and short confusion assessment method. Psychosomatics. 2019;60:172–8. 31416628 10.1016/j.psym.2018.09.002

[14] Brattinga B Plas M Spikman JM. The association between the inflammatory response following surgery and post-operative delirium in older oncological patients: a prospective cohort study. Age Ageing. 2022;51:1–9. 10.1093/ageing/afab237 PMC9160877 35180288

[15] Adamis D Slor CJ Leonard M. Reliability of delirium rating scale (DRS) and delirium rating scale-revised-98 (DRS-R98) using variance-based multivariate modelling. J Psychiatr Res. 2013;47:966–71. 23522935 10.1016/j.jpsychires.2013.02.012

[16] Park J Jeong E Lee J. The delirium observation screening scale: a systematic review and meta-analysis of diagnostic test accuracy. Clin Nurs Res. 2021;30:464–73. 33174438 10.1177/1054773820961234

[17] Dormandy L Mufti S Higgins E. Shifting the focus: a QI project to improve the management of delirium in patients with hip fracture. Future Healthc J. 2019;6:215–9. 31660529 10.7861/fhj.2019-0006 PMC6798014

[18] Maldonado JR Sher YI Benitez-Lopez MA. A study of the psychometric properties of the “stanford proxy test for delirium” (S-PTD): a new screening tool for the detection of delirium. Psychosomatics. 2020;61:116–26. 31926650 10.1016/j.psym.2019.11.009

[19] Evered LA Chan MTV Han R. Anaesthetic depth and delirium after major surgery: a randomised clinical trial. Br J Anaesth. 2021;127:704–12. 34465469 10.1016/j.bja.2021.07.021 PMC8579421

[20] Valentin LS Pereira VF Pietrobon RS. Effects of single low dose of dexamethasone before noncardiac and nonneurologic surgery and general anesthesia on postoperative cognitive dysfunction-a phase III double blind, randomized clinical trial. PLoS One. 2016;11:e0152308. 27152422 10.1371/journal.pone.0152308 PMC4859565

[21] Peng M Wang YL Wang FF. The cyclooxygenase-2 inhibitor parecoxib inhibits surgery-induced proinflammatory cytokine expression in the hippocampus in aged rats. J Surg Res. 2012;178:e1–8. 22959208 10.1016/j.jss.2012.08.030

[22] Zhao WX Zhang JH Cao JB. Acetaminophen attenuates lipopolysaccharide-induced cognitive impairment through antioxidant activity. J Neuroinflammation. 2017;14:17. 28109286 10.1186/s12974-016-0781-6 PMC5251335

[23] Alam A Hana Z Jin Z. Surgery, neuroinflammation and cognitive impairment. EBioMedicine. 2018;37:547–56. 30348620 10.1016/j.ebiom.2018.10.021 PMC6284418

[24] Li K Wang J Chen L. Netrin-1 ameliorates postoperative delirium-like behavior in aged mice by suppressing neuroinflammation and restoring impaired blood-brain barrier permeability. Front Mol Neurosci. 2021;14:751570. 35095412 10.3389/fnmol.2021.751570 PMC8797926

[25] Ni P Dong H Wang Y. IL-17A contributes to perioperative neurocognitive disorders through blood-brain barrier disruption in aged mice. J Neuroinflammation. 2018;15:332. 30501622 10.1186/s12974-018-1374-3 PMC6267879

[26] Pan S Lv Z Wang R. Sepsis-induced brain dysfunction: pathogenesis, diagnosis, and treatment. Oxid Med Cell Longev. 2022;2022:1328729. 36062193 10.1155/2022/1328729 PMC9433216

[27] Hasegawa-Ishii S Inaba M Umegaki H. Endotoxemia-induced cytokine-mediated responses of hippocampal astrocytes transmitted by cells of the brain-immune interface. Sci Rep. 2016;6:25457. 27149601 10.1038/srep25457 PMC4857737

[28] Stubbs DJ Yamamoto AK Menon DK. Imaging in sepsis-associated encephalopathy--insights and opportunities. Nat Rev Neurol. 2013;9:551–61. 23999468 10.1038/nrneurol.2013.177

[29] Zhou HC Guo CA Yu WW. Zizyphus jujuba cv. Muzao polysaccharides enhance intestinal barrier function and improve the survival of septic mice. J Food Biochem. 2021;45:e13722. 33855723 10.1111/jfbc.13722

[30] Yu L Wen G Zhu S. Abnormal phosphorylation of tau protein and neuroinflammation induced by laparotomy in an animal model of postoperative delirium. Exp Brain Res. 2021;239:867–80. 33409674 10.1007/s00221-020-06007-2

[31] Cheon SY Koo BN Kim SY. Scopolamine promotes neuroinflammation and delirium-like neuropsychiatric disorder in mice. Sci Rep. 2021;11:8376. 33863952 10.1038/s41598-021-87790-y PMC8052461

[32] Qin L Wu X Block ML. Systemic LPS causes chronic neuroinflammation and progressive neurodegeneration. Glia. 2007;55:453–62. 17203472 10.1002/glia.20467 PMC2871685

[33] Batista CRA Gomes GF Candelario-Jalil E. Lipopolysaccharide-induced neuroinflammation as a bridge to understand neurodegeneration. Int J Mol Sci . 2019;20:2293. 31075861 10.3390/ijms20092293 PMC6539529

[34] Silva SP Zago AM Carvalho FB. Neuroprotective effect of taurine against cell death, glial changes, and neuronal loss in the cerebellum of rats exposed to chronic-recurrent neuroinflammation induced by LPS. J Immunol Res. 2021;2021:7497185. 34327244 10.1155/2021/7497185 PMC8277510

[35] Huffman WJ Subramaniyan S Rodriguiz RM. Modulation of neuroinflammation and memory dysfunction using percutaneous vagus nerve stimulation in mice. Brain Stimul. 2019;12:19–29. 30337243 10.1016/j.brs.2018.10.005 PMC6301148

[36] Milner MT Maddugoda M Gotz J. The NLRP3 inflammasome triggers sterile neuroinflammation and Alzheimer’s disease. Curr Opin Immunol. 2021;68:116–24. 33181351 10.1016/j.coi.2020.10.011

[37] Lopez-Rodriguez AB Hennessy E Murray CL. Acute systemic inflammation exacerbates neuroinflammation in Alzheimer’s disease: IL-1beta drives amplified responses in primed astrocytes and neuronal network dysfunction. Alzheimers Dement. 2021;17:1735–55. 34080771 10.1002/alz.12341 PMC8874214

[38] Skelly DT Griffin EW Murray CL. Acute transient cognitive dysfunction and acute brain injury induced by systemic inflammation occur by dissociable IL-1-dependent mechanisms. Mol Psychiatry. 2019;24:1533–48. 29875474 10.1038/s41380-018-0075-8 PMC6510649

[39] Hoover DB. Cholinergic modulation of the immune system presents new approaches for treating inflammation. Pharmacol Ther. 2017;179:1–16. 28529069 10.1016/j.pharmthera.2017.05.002 PMC5651192

[40] Kim YS Choi J Yoon BE. Neuron-glia interactions in neurodevelopmental disorders. Cells. 2020;9:2176. 32992620 10.3390/cells9102176 PMC7601502

[41] Tang Y Le W. Differential roles of M1 and M2 microglia in neurodegenerative diseases. Mol Neurobiol. 2016;53:1181–94. 25598354 10.1007/s12035-014-9070-5

[42] Block ML Zecca L Hong JS. Microglia-mediated neurotoxicity: uncovering the molecular mechanisms. Nat Rev Neurosci. 2007;8:57–69. 17180163 10.1038/nrn2038

[43] Colton CA. Heterogeneity of microglial activation in the innate immune response in the brain. J Neuroimmune Pharmacol. 2009;4:399–418. 19655259 10.1007/s11481-009-9164-4 PMC2773116

[44] Yang T Velagapudi R Terrando N. Neuroinflammation after surgery: from mechanisms to therapeutic targets. Nat Immunol. 2020;21:1319–26. 33077953 10.1038/s41590-020-00812-1 PMC7704062

[45] Lv S Song HL Zhou Y. Tumour necrosis factor-alpha affects blood-brain barrier permeability and tight junction-associated occludin in acute liver failure. Liver Int. 2010;30:1198–210. 20492508 10.1111/j.1478-3231.2010.02211.x

[46] Xue J Suarez JS Minaai M. HMGB1 as a therapeutic target in disease. J Cell Physiol. 2021;236:3406–19. 33107103 10.1002/jcp.30125 PMC8104204

[47] Tan SW Zhao Y Li P. HMGB1 mediates cognitive impairment caused by the NLRP3 inflammasome in the late stage of traumatic brain injury. J Neuroinflammation. 2021;18:241. 34666797 10.1186/s12974-021-02274-0 PMC8527642

[48] Wang ML Min J Sands LP. Midazolam premedication immediately before surgery is not associated with early postoperative delirium. Anesth Analg. 2021;133:765–71. 33721875 10.1213/ANE.0000000000005482 PMC8373629

[49] Rump K Holtkamp C Bergmann L. Midazolam impacts acetyl-And butyrylcholinesterase genes: an epigenetic explanation for postoperative delirium? PLoS One. 2022;17:e0271119. 35802656 10.1371/journal.pone.0271119 PMC9269431

[50] Yang M Liu X Yang D. Effect of remimazolam besylate compared with propofol on the incidence of delirium after cardiac surgery: study protocol for a randomized trial. Trials. 2021;22:717. 34663423 10.1186/s13063-021-05691-x PMC8522864

[51] Liu X Lin S Zhong Y. Remimazolam protects against LPS-induced endotoxicity improving survival of endotoxemia mice. Front Pharmacol. 2021;12:739603. 34867346 10.3389/fphar.2021.739603 PMC8641375

[52] Zhou XH Zhang CC Wang L. Remimazolam induced cognitive dysfunction in mice via glutamate excitotoxicity. Transl Neurosci. 2022;13:104–15. 35734308 10.1515/tnsci-2022-0220 PMC9164290

[53] Liu X Zhang K Wang W. Dexmedetomidine sedation reduces atrial fibrillation after cardiac surgery compared to propofol: a randomized controlled trial. Crit Care. 2016;20:298. 27654700 10.1186/s13054-016-1480-5 PMC5031329

[54] Li T Li J Yuan L. Effect of regional vs general anesthesia on incidence of postoperative delirium in older patients undergoing hip fracture surgery: the RAGA randomized trial. JAMA. 2022;327:50–8. 34928310 10.1001/jama.2021.22647 PMC8689436

[55] De Simone R Niturad CE De Nuccio C. TGF-beta and LPS modulate ADP-induced migration of microglial cells through P2Y1 and P2Y12 receptor expression. J Neurochem. 2010;115:450–9. 20681951 10.1111/j.1471-4159.2010.06937.x

[56] Tang C Hu Y Zhang Z. Dexmedetomidine with sufentanil in intravenous patient-controlled analgesia for relief from postoperative pain, inflammation and delirium after esophageal cancer surgery. Biosci Rep. 2020;40:BSR20193410. 32343308 10.1042/BSR20193410 PMC7214400

[57] Kadry H Noorani B Cucullo L. A blood-brain barrier overview on structure, function, impairment, and biomarkers of integrity. Fluids Barriers CNS. 2020;17:69. 33208141 10.1186/s12987-020-00230-3 PMC7672931

[58] Taylor J Parker M Casey CP. Postoperative delirium and changes in the blood-brain barrier, neuroinflammation, and cerebrospinal fluid lactate: a prospective cohort study. Br J Anaesth. 2022;129:219–30. 35144802 10.1016/j.bja.2022.01.005 PMC9465948

[59] Matsuda M Huh Y Ji RR. Roles of inflammation, neurogenic inflammation, and neuroinflammation in pain. J Anesth. 2019;33:131–9. 30448975 10.1007/s00540-018-2579-4 PMC6813778

[60] Yang S Gu C Mandeville ET. Anesthesia and surgery impair blood-brain barrier and cognitive function in mice. Front Immunol. 2017;8:902. 28848542 10.3389/fimmu.2017.00902 PMC5552714

[61] Roth TL Nayak D Atanasijevic T. Transcranial amelioration of inflammation and cell death after brain injury. Nature. 2014;505:223–8. 24317693 10.1038/nature12808 PMC3930079

[62] Tang Y Li T Li J. Jmjd3 is essential for the epigenetic modulation of microglia phenotypes in the immune pathogenesis of Parkinson’s disease. Cell Death Differ. 2014;21:369–80. 24212761 10.1038/cdd.2013.159 PMC3921590

[63] Zhou X Spittau B Krieglstein K. TGFβ signalling plays an important role in IL4-induced alternative activation of microglia. J Neuroinflammation. 2012;9:210. 22947253 10.1186/1742-2094-9-210 PMC3488564

[64] Wan Y Feng B You Y. Microglial displacement of GABAergic synapses is a protective event during complex febrile seizures. Cell Rep. 2020;33:108346. 33147450 10.1016/j.celrep.2020.108346

[65] Sofroniew MV. Astrocyte barriers to neurotoxic inflammation. Nat Rev Neurosci. 2015;16:249–63. 25891508 10.1038/nrn3898 PMC5253239

[66] Notter T Coughlin JM Gschwind T. Translational evaluation of translocator protein as a marker of neuroinflammation in schizophrenia. Mol Psychiatry. 2018;23:323–34. 28093569 10.1038/mp.2016.248

[67] Hu MH Zheng QF Jia XZ. Neuroprotection effect of interleukin (IL)-17 secreted by reactive astrocytes is emerged from a high-level IL-17-containing environment during acute neuroinflammation. Clin Exp Immunol. 2014;175:268–84. 24117055 10.1111/cei.12219 PMC3892418

[68] Brigas HC Ribeiro M Coelho JE. IL-17 triggers the onset of cognitive and synaptic deficits in early stages of Alzheimer’s disease. Cell Rep. 2021;36:109574. 34469732 10.1016/j.celrep.2021.109574

[69] Henjum K Quist-Paulsen E Zetterberg H. CSF sTREM2 in delirium-relation to Alzheimer’s disease CSF biomarkers Aβ42, t-tau and p-tau. J Neuroinflammation. 2018;15:304. 30390679 10.1186/s12974-018-1331-1 PMC6215363

[70] Beishuizen SJ Scholtens RM Van Munster B C. Unraveling the relationship between delirium, brain damage, and subsequent cognitive decline in a cohort of individuals undergoing surgery for hip fracture. J Am Geriatr Soc. 2017;65:130–6. 27641367 10.1111/jgs.14470

[71] Forsberg A Cervenka S Jonsson Fagerlund M. The immune response of the human brain to abdominal surgery. Ann Neurol. 2017;81:572–82. 28253549 10.1002/ana.24909 PMC12900553

[72] Al Tmimi L Van De Velde M Meyns B. Serum protein S100 as marker of postoperative delirium after off-pump coronary artery bypass surgery: secondary analysis of two prospective randomized controlled trials. Clin Chem Lab Med. 2016;54:1671–80. 26943607 10.1515/cclm-2015-1012

[73] Liu P Li YW Wang XS. High serum interleukin-6 level is associated with increased risk of delirium in elderly patients after noncardiac surgery: a prospective cohort study. Chin Med J (Engl). 2013;126:3621–7. 24112153

[74] Harmon D Eustace N Ghori K. Plasma concentrations of nitric oxide products and cognitive dysfunction following coronary artery bypass surgery. Eur J Anaesthesiol. 2005;22:269–76. 15892404 10.1017/s0265021505000451

[75] Androsova G Krause R Winterer G. Biomarkers of postoperative delirium and cognitive dysfunction. Front Aging Neurosci. 2015;7:112. 26106326 10.3389/fnagi.2015.00112 PMC4460425

[76] Nguyen PT Dorman LC Pan S. Microglial remodeling of the extracellular matrix promotes synapse plasticity. Cell. 2020;182:388–403.e15. 32615087 10.1016/j.cell.2020.05.050 PMC7497728

[77] Ribeiro M Brigas HC Temido-Ferreira M. Meningeal gammadelta T cell-derived IL-17 controls synaptic plasticity and short-term memory. Sci Immunol. 2019;4:eaay5199. 31604844 10.1126/sciimmunol.aay5199 PMC6894940

[78] Park GH Noh H Shao Z. Activated microglia cause metabolic disruptions in developmental cortical interneurons that persist in interneurons from individuals with schizophrenia. Nat Neurosci. 2020;23:1352–64. 33097921 10.1038/s41593-020-00724-1 PMC7769122

[79] Wang L Hauenstein AV. The NLRP3 inflammasome: Mechanism of action, role in disease and therapies. Mol Aspects Med. 2020;76:100889. 32859386 10.1016/j.mam.2020.100889

[80] Shi M Chen J Liu T. Protective effects of remimazolam on cerebral ischemia/reperfusion injury in rats by inhibiting of NLRP3 inflammasome-dependent pyroptosis. Drug Des Devel Ther. 2022;16:413–23. 10.2147/DDDT.S344240 PMC8863189 35210755

[81] Wyrobek J LaFlam A Max L. Association of intraoperative changes in brain-derived neurotrophic factor and postoperative delirium in older adults. Br J Anaesth. 2017;119:324–32. 28854532 10.1093/bja/aex103 PMC6172970

[82] Travica N Aslam H O’Neil A. Brain derived neurotrophic factor in perioperative neurocognitive disorders: current evidence and future directions. Neurobiol Learn Mem. 2022;193:107656. 35792324 10.1016/j.nlm.2022.107656

[83] Mo M Eyo UB Xie M. Microglial P2Y12 receptor regulates seizure-induced neurogenesis and immature neuronal projections. J Neurosci. 2019;39:9453–64. 31597724 10.1523/JNEUROSCI.0487-19.2019 PMC6867812

[84] Song WM Colonna M. The identity and function of microglia in neurodegeneration. Nat Immunol. 2018;19:1048–58. 30250185 10.1038/s41590-018-0212-1

[85] Eyo UB Mo M Yi M-H. P2Y12R-dependent translocation mechanisms gate the changing microglial landscape. Cell Rep. 2018;23:959–66. 29694903 10.1016/j.celrep.2018.04.001 PMC5965271

[86] Lou N Takano T Pei Y. Purinergic receptor P2RY12-dependent microglial closure of the injured blood-brain barrier. Proc Natl Acad Sci U S A. 2016;113:1074–9. 26755608 10.1073/pnas.1520398113 PMC4743790

[87] Lazarević M Golubović M Milić D. Preoperative levels of the soluble urokinase-type plasminogen activator receptor as predictor for new episodes of atrial fibrillation after vascular surgery. Vasc Endovascular Surg. 2021;55:461–6. 33622185 10.1177/1538574421995321

[88] Herroeder S Pecher S Schönherr ME. Systemic lidocaine shortens length of hospital stay after colorectal surgery: a double-blinded, randomized, placebo-controlled trial. Ann Surg. 2007;246:192–200. 17667496 10.1097/SLA.0b013e31805dac11 PMC1933564

[89] Rump K Adamzik M. Epigenetic mechanisms of postoperative cognitive impairment induced by anesthesia and neuroinflammation. Cells. 2022;11:2954. 36230916 10.3390/cells11192954 PMC9563723

[90] Zhang L Xue Z Liu Q. Disrupted folate metabolism with anesthesia leads to myelination deficits mediated by epigenetic regulation of ERMN. EBioMedicine. 2019;43:473–86. 31060905 10.1016/j.ebiom.2019.04.048 PMC6562069

[91] Garden GA. Epigenetics and the modulation of neuroinflammation. Neurotherapeutics. 2013;10:782–8. 23963788 10.1007/s13311-013-0207-4 PMC3805872

[92] Lin J Wang S Feng Y. Propofol exposure during early gestation impairs learning and memory in rat offspring by inhibiting the acetylation of histone. J Cell Mol Med. 2018;22:2600–11. 29461008 10.1111/jcmm.13524 PMC5908131

[93] Yang L Hao JR Gao Y. HDAC3 of dorsal hippocampus induces postoperative cognitive dysfunction in aged mice. Behav Brain Res. 2022;433:114002. 35810999 10.1016/j.bbr.2022.114002

[94] Dudek KA Dion-Albert L Lebel M. Molecular adaptations of the blood-brain barrier promote stress resilience vs. depression. Proc Natl Acad Sci U S A. 2020;117:3326–36. 31974313 10.1073/pnas.1914655117 PMC7022213

[95] Kvichansky AA Volobueva MN Spivak YS. Expression of mRNAs for IL-1β, IL-6, IL-10, TNFα, CX3CL1, and TGFβ1 cytokines in the brain tissues: assessment of contribution of blood cells with and without perfusion. Biochemistry (Mosc). 2019;84:905–10. 31522672 10.1134/S0006297919080066

[96] Hüls A Robins C Conneely KN. Brain DNA methylation patterns in CLDN5 associated with cognitive decline. Biol Psychiatry. 2022;91:389–98. 33838873 10.1016/j.biopsych.2021.01.015 PMC8329105

[97] Yang F Zhao K Zhang X. ATP Induces disruption of tight junction proteins via IL-1 Beta-Dependent MMP-9 activation of human blood-brain barrier in vitro. Neural Plast. 2016;2016:8928530. 27795859 10.1155/2016/8928530 PMC5067334

[98] Zhang Y Ding X Miao C. Propofol attenuated TNF-α-modulated occludin expression by inhibiting Hif-1α/ VEGF/ VEGFR-2/ ERK signaling pathway in hCMEC/D3 cells. BMC Anesthesiol. 2019;19:127. 31288745 10.1186/s12871-019-0788-5 PMC6617648

